# Modified bio-Bentall operation with a rapid deployment valve

**DOI:** 10.1016/j.xjtc.2025.04.003

**Published:** 2025-04-30

**Authors:** Hyo Kyen Park, Hong Rae Kim, Joon Bum Kim

**Affiliations:** Department of Thoracic and Cardiovascular Surgery, Asan Medical Center, University of Ulsan College of Medicine, Seoul, Republic of Korea

**Keywords:** Bentall operation, rapid deployment valve, paravalvular leakage

## Abstract

**Objective:**

To evaluate the early outcomes of a modified bio-Bentall operation using a rapid deployment valve (RDV) in high-risk patients, focusing on procedural efficacy, survival rates, and complications.

**Methods:**

A retrospective review of 11 consecutive patients who underwent the modified bio-Bentall operation with an RDV between January 2018 and December 2022 was conducted. Kaplan–Meier survival analysis was used to determine survival rates. Patients' baseline characteristics, operative details, and postoperative outcomes were reviewed.

**Results:**

The median patient age was 71 years. Most patients presenting with high-risk conditions and significant comorbidities, including inflammatory conditions, chronic lung diseases, and advanced cardiac dysfunction. The median EuroSCORE II was 10.03%. The median aortic cross-clamping and cardiopulmonary bypass times were 73.0 minutes and 99.0 minutes, respectively. No early mortalities or reoperations occurred. Two patients (14.3%) required extracorporeal membrane oxygenation support because of low cardiac output and arrhythmias, and 3 patients (23.1%) required permanent pacemaker insertion. No paravalvular leakage or valve detachment was observed during follow-up. The 1-year survival rate was 90.9%, and the 3-year survival rate was 54.5%. Six patients died during a median follow-up of 35 months, from causes including respiratory complications, gastric cancer, and undetermined factors.

**Conclusions:**

Our initial experience with the modified bio-Bentall operation using an RDV shows favorable early outcomes in relatively high-risk patients. Further validation with larger datasets and long-term follow-up is needed to validate these results.

**Figure:**
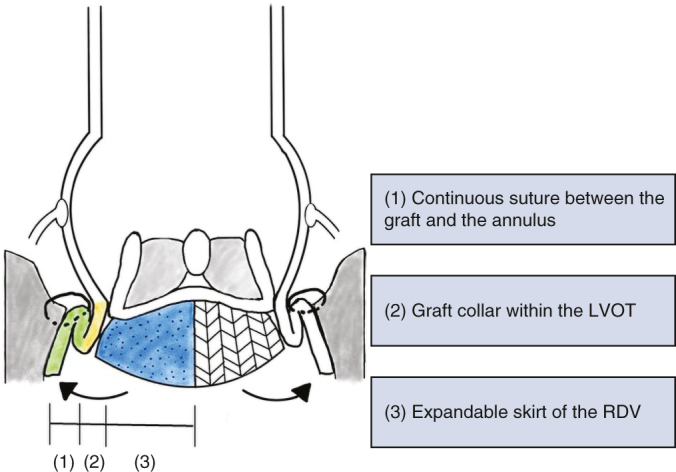
Multiple hemostatic layers aim to provide stability and potentially reduce complications.

**Central Message**

The modified bio-Bentall operation with a rapid deployment valve showed favorable early outcomes in high-risk patients.

**Perspective**

This study evaluating the early outcomes of a modified bio-Bentall operation with a rapid deployment valve in high-risk patients suggests favorable outcomes, including reduced surgical times and complications, indicating that this approach may offer potential benefits in the management of high-risk aortic root replacement surgeries.

Composite mechanical or bioprosthetic valve grafts has become the standard approach for complete aortic root replacement.^1^^,^^2^ However, conditions such as vasculitis syndromes, including cardiac Behçet disease, and infective endocarditis often involve fragile and inflamed tissues, which pose a significant challenge when performing valve implantation using single-layer horizontal mattress sutures. These pathologic conditions markedly increase the risk of complications, such as bleeding, valve detachment, and pseudoaneurysm formation.^3^^,^^4^

To mitigate such risks, we adopted a modified technique, incorporating the implantation of an INTUITY valve (Edwards Lifesciences), followed by direct anastomosis of a Valsalva graft (Terumo Medical) to the left ventricular outflow tract (LVOT).^5^ This approach aimed to achieve multilayer hemostasis through enhanced stability and rapid execution of the procedure, particularly in high-risk patients. We initially performed this modified Bentall operation for high-risk conditions only, but its application has since expanded to average-risk groups. Here we report the early outcomes associated with this approach.

## Methods

### Patients

We identified consecutive patients who underwent a modified bio-Bentall operation with an INTUITY rapid deployment valve (RDV) between January 2018 and December 2022. We initially used this procedure in patients with cardiac Behçet disease, who were at high risk for annular dehiscence. With accumulating experience, we have come to perceive the procedure as not only expediting the surgical process, but also conferring significant hemostatic advantages. Consequently, its application was expanded to include a broader range of patients. A retrospective review of these patients was conducted subsequently. The study was approved by the Institutional Ethics Committee/Review Board of the Asan Medical Center (2025-0105; approved January 22, 2025), and the requirement for informed patient consent was waived in view of the study's retrospective nature.

### Operative Procedure

The surgical approach typically involved an upper partial sternotomy through the third or fourth intercostal space. In cases requiring redo surgeries or concomitant procedures such as coronary artery bypass grafting (CABG) or mitral valve repair, a full sternotomy was performed. For patients undergoing root surgery alone, normothermic cardiac arrest was the primary strategy. For those requiring concomitant aortic arch surgery, moderate hypothermic circulatory arrest with a target nasopharyngeal temperature of 25 to 28 °C was used, with or without unilateral antegrade cerebral perfusion, depending on the extent of the arch repair.^6^

**Figure 1:**
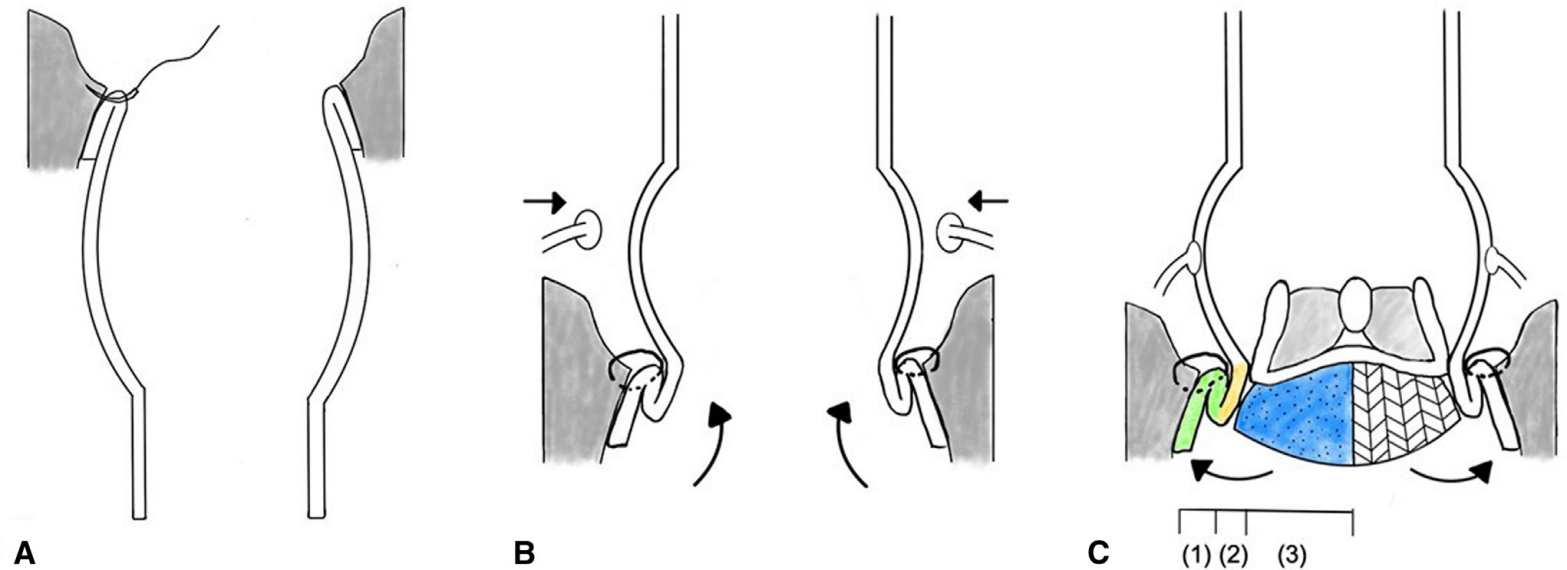
Operative schema. A, The graft was inverted within the left ventricular outflow tract (*LVOT*) to ensure optimal visibility for performing a single-layer continuous suture. B, The graft was exteriorized, with initial suturing of the coronary buttons performed first, followed by the insertion of the valve. C, Between the LVOT and the aortic graft was easily secured by 3 hemostatic barriers: (1) anchoring continuous suture between the Valsalva graft and annulus, (2) collar of the Valsalva in the LVOT, and (3) rapid deployment valve skirt.

**Figure 2:**
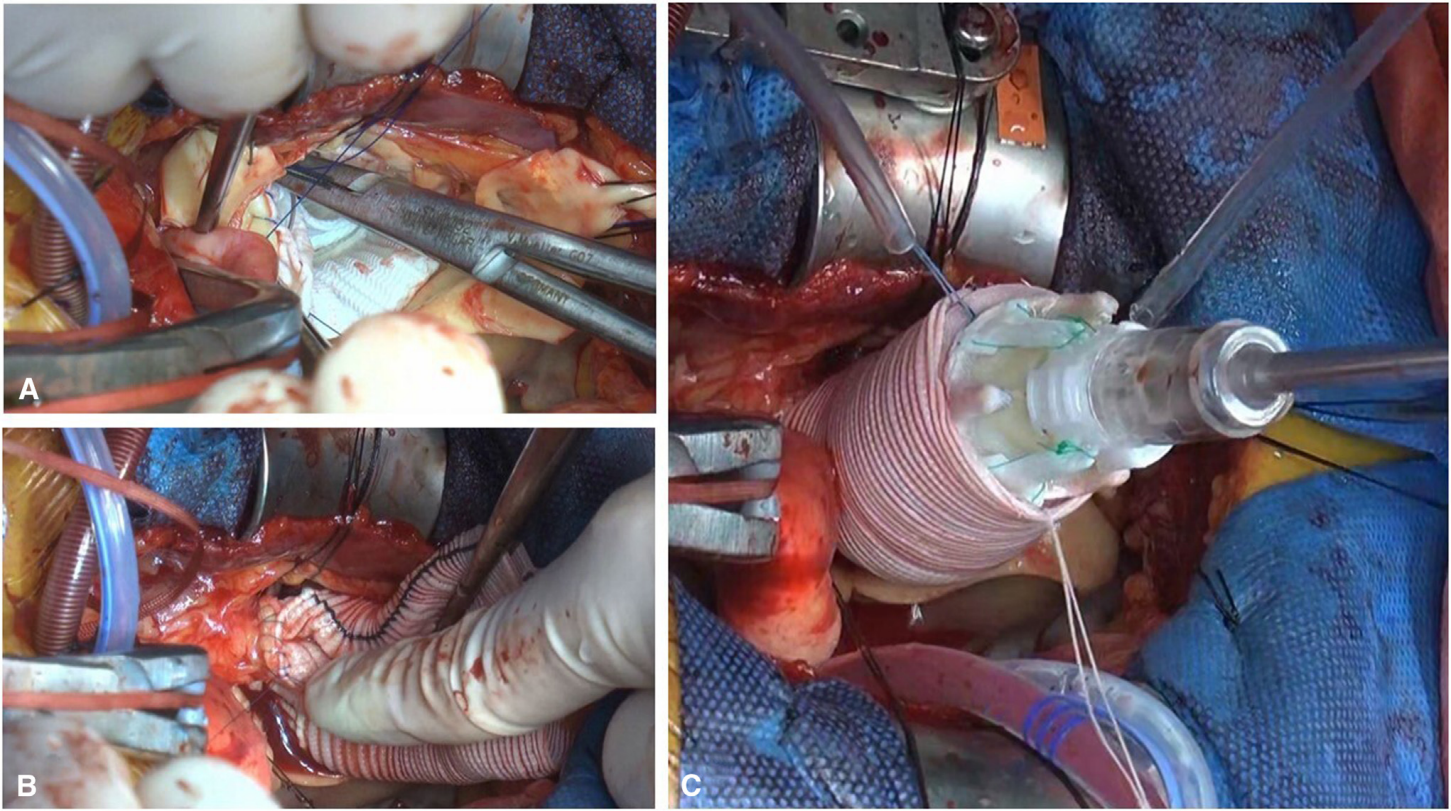
Modified bio-Bentall operation. A, 30-mm Valsalva graft cut to 8-cm length was inverted and inserted into the left ventricular outflow tract. B, Coronary buttons were reimplanted. C, An INTUITY valve was inserted using the suture as a guide and carefully deployed.

After aortic cross-clamping, 1 L of antegrade del Nido cardioplegic solution was administered either through the aortic root or directly into the coronary ostia, depending on the presence of aortic insufficiency (AI). The ascending aorta and aortic root were excised down to the level of the aortic valve annulus, and coronary buttons were prepared. The size of the Valsalva graft was based on the dimensions of the aortic annulus. The graft was prepared by cutting it to a length of 8 cm, after which it was inverted, folded, and inserted into the LVOT. Proximal anastomosis was performed using a single-layer continuous suture with 3-0 polypropylene (Figure 1, *A*). After this stage, the valve size was measured, and the largest suitable size was prepared. Then the graft was exteriorized, and the coronary buttons were reimplanted (Figure 1, *B*) using 5-0 polypropylene. Following this, 3 equidistant RDV anchoring sutures with 2-0 braided polyester, in a simple interrupted manner, were placed at the nadir parts of the graft. An INTUITY valve was guided into position using these anchoring sutures and carefully deployed (Figure 2). The deployment depth was assessed to ensure that the expandable frame was seated below the proximal edge of the graft (Figure 1, *C*). Proper seating was confirmed both visually and by gently retracting the guiding sutures to achieve symmetrical expansion before final deployment. Finally, the distal anastomosis was completed (Video 1).

### Statistical Analysis

Values are presented as median with range for continuous variables. Categorical variables are shown as total number and proportion in percentage. The Kaplan-Meier method was used to analyze the survival rates. All statistical analyses were performed using R version 4.2.2 (R Foundation for Statistical Computing).

## Results

### Baseline Characteristics

During the study period, 11 patients underwent the modified bio-Bentall technique with an RDV. Patient characteristics and operative profiles are summarized in Table 1. The median patient age was 71 years (range, 63-88 years), and 7 patients (63.8%) were male. The primary indications for surgery included annuloaortic ectasia with AI in 6 patients (54.5%), aortic dissection with severe AI in 2 patients (18.2%), infective endocarditis of the aortic valve in 2 patients (18.2%), and cardiac Behçet disease in 1 patient (9.1%). One patient (9.1%) had a history of prior hemiarch replacement owing to acute type A aortic dissection and later presented with residual root dissection with severe AI. The median ejection fraction (EF) was 46% (range, 28%-60 %) and the median glomerular filtration rate was 42 mL/min/1.73 mm^2^ (range, 12-92 mL/min/1.73 mm^2^). The median EuroSCORE II was 10.03% (range, 2.52%-62.72 %), with 4 patients classified as intermediate risk (8 > EuroSCORE II ≥ 2) and 7 patients classified as high risk (EuroSCORE II ≥ 8). Three patients presented with thrombocytopenia, with etiology attributed to autoimmune hemolytic anemia, liver cirrhosis, and an unknown origin, respectively.

**Table 1:** Patient characteristics and operative profiles (N = 11)

Patient	Diagnosis	Age, y	Sex	DM	GFR (mL/min/1.73 mm^2^)	Thrombocytopenia	EF, %	Concomitant procedure	EuroSCORE II, %
1	Chronic type A AD	75	F	-	38	Yes	60	Total arch replacement	35.0
2	Behçet's disease, 2VD	63	F	Yes	36	-	35	Hemiarch replacement, CABG	62.7
3	Acute type A AD	88	F	-	42	-	42	Hemiarch replacement	56.0
4	AAE, severe MR, severe TR	67	F	-	24	-	28	Mitral valve repair	35.2
5	AAE	75	M	-	39	-	45	Hemiarch replacement	10.0
6	AAE	64	M	-	57	-	35	Hemiarch replacement	4.5
7	Infective endocarditis	71	M	-	92	Yes	47	LVOT reconstruction	8.2
8	AAE, moderate MR	79	M	-	57	-	46	Mitral valve repair	5.6
9	Infective endocarditis	68	M	Yes	12	Yes	57	-	20.4
10	AAE	69	M	-	69	-	48	-	2.5
11	AAE	75	M	-	63	-	46	-	3.6

*DM*, Diabetes mellitus; *GFR*, glomerular filtration rate; *EF*, ejection fraction; *EuroSCORE II*, European System for Cardiac Operative Risk Evaluation II; *AD*, aortic dissection; *2VD*, 2 vessel disease; *CABG*, coronary artery bypass grafting; *AAE*, annuloaortic ectasia; *MR*, mitral regurgitation; *TR*, tricuspid regurgitation; *LVOT*, left ventricular outflow tract.

### Operative Characteristics

The procedure was performed electively in 6 patients (54.5%) and urgently or emergently in 5 patients (45.5%) (Table 2). Eight patients required concomitant procedures in addition to the modified bio-Bentall operation, with aortic arch surgery—comprising hemiarch replacement (27.3%)—the most common. Among 6 patients undergoing concomitant arch repair, 5 underwent upper sternotomy and 1 underwent emergent surgery for acute type II aortic dissection via full sternotomy. In cases involving other concomitant procedures (n = 5), a full sternotomy was performed. The median size of the RDV used in patients was 23 mm (range, 19-27 mm), which was approximately 5 to 7 mm smaller than the graft size. The median aortic cross-clamping and cardiopulmonary bypass times were 73.0 minutes (range, 46-154 minutes) and 99.0 minutes (range, 62-204 minutes), respectively. In particular, for isolated Bentall operations (n = 3), all of which were performed via upper sternotomy, median aortic cross-clamping and cardiopulmonary bypass times were 62.0 minutes (range, 46-73 minutes) and 88.0 minutes (range, 62-99 minutes), respectively.

**Table 2:** Operative variables (N = 11)

Variable	Value
Approach, n (%)	
Full sternotomy	6 (54.5)
Upper sternotomy (third ICS)	3 (27.3)
Upper sternotomy (fourth ICS)	2 (18.2)
Concomitant operation, n (%)	
Hemiarch replacement	3 (27.3)
Total arch replacement	1 (9.1)
Mitral valve repair	2 (18.2)
LVOT reconstruction	1 (9.1)
Hemiarch replacement + CABG	1 (9.1)
Urgency, n (%)	
Elective	6 (54.5)
Urgent	4 (36.4)
Emergency	1 (9.1)
Procedure time, median (range)	
Aortic cross-clamp time, min	73 (46-154)
Cardiopulmonary bypass time, min	99 (62-204)
Entire procedure time, min	272 (272-474)

*ICS*, Intercostal space; *LVOT*, left ventricular outflow tract; *CABG*, coronary artery bypass grafting.

### Outcomes

There were no early mortalities or reoperations due to bleeding, and no cases of paravalvular leakage (PVL) were observed during the postoperative follow-up period. Two patients (14.3%) required extracorporeal membrane oxygenation (ECMO) support. Of these, 1 patient with cardiac Behçet disease who had been receiving high-dose inotropic support preoperatively for severe left ventricular dysfunction developed low cardiac output syndrome immediately following surgery, necessitating ECMO. The other patient, a 75-year-old with chronic kidney disease and an EF of 45%, experienced ventricular fibrillation arrest on postoperative day 9 and also required ECMO support. Three patients (23.1%) required permanent pacemaker (PPM) insertion, including 2 patients who developed complete atrioventricular block (AVB) postoperatively and 1 patient with postoperative tachy-brady syndrome.

One patient, who had undergone hemiarch replacement for acute type A aortic dissection 2 years earlier, presented with residual dissection in the aortic root and severe AI and underwent a modified bio-Bentall operation and total arch replacement. Postoperatively, the patient experienced a seizure, and subsequent imaging revealed multiple embolic infarcts; however, they recovered without neurologic deficits (Table 3).

**Table 3:** Early outcomes of the patient population (N = 11)

Outcome	Value
Early death, n	0
Postoperative complications, n (%)	
Disabling neurologic event	0
Nondisabling neurologic event	1 (7.7)
Reexploration for bleeding control	0
Prolonged ventilator support (>48 h)	2 (15.4)
Postoperative pneumonia	1 (7.7)
Extracorporeal membrane oxygenation	2 (15.4)
Permanent pacemaker insertion	3 (23.1)
Length of stay, d, median (range)	
Intensive care unit stay	3 (1-26)
Postoperative hospitalization stay	22 (6-54)

During a median follow-up of 35 months (range, 24-82 months), 6 deaths occurred, with causes including respiratory complications in 3 patients, gastric cancer in 1 patient, and unknown causes in 2 patients. Three patients with chronic lung disease (forced expiratory volume in 1 second values of 60%, 63%, and 54%) later developed pneumonia and died at 10, 13, and 18 months after discharge. Another patient who was discharged without any acute complications developed gastric cancer 2 years later and died during chemotherapy. The remaining 2 patients died at 15 and 19 months after discharge, both from unknown causes. One of these patients, who underwent urgent surgery for cardiac Behçet disease, presented with multivessel coronary disease and severe congestive heart failure necessitating concomitant CABG during the index surgery. The other patient, who was diagnosed with annuloaortic ectasia, severe AI, and moderate MR and had an EF of 46%, underwent MV repair as a concomitant procedure. This patient, who was also receiving adjuvant chemotherapy for prostate cancer, had an uneventful postoperative course but later died from causes that remain undetermined.

The overall survival rate was 90.9% (95% confidence interval, 75.4%-100%) at 1 year and 54.5% (95% confidence interval, 31.8%-93.6%) at 3 years. No reoperations or valve-related complications occurred during the follow-up period.

## Discussion

This study evaluated the outcomes of the modified bio-Bentall operation in intermediate- and high-risk patients, the majority of whom had significant comorbidities, including inflammatory conditions, chronic lung diseases, and advanced cardiac dysfunction. The modified procedure, which incorporates an RDV and multiple hemostatic layers constructed by a single-layer anastomosis, was intended to provide stability, to potentially reduce complications such as valve detachment and bleeding, and to possibly expedite the surgical process. Despite the severity of their preoperative conditions, the favorable early outcomes observed in this patient group, including a short aortic cross-clamping time and the absence of operative mortality, reoperations, or bleeding, suggest potential advantages of this approach for high-risk individuals.

The procedure appears to achieve a robust root anastomosis through 3 key features: (1) an anchoring continuous suture between the graft and the annulus, (2) the positioning of the graft collar within the left ventricular outflow tract, and (3) the expandable skirt of the RDV (Figure 1). These features aim to provide structural support, potentially contributing to stability in cases of tissue fragility. This modification also may contribute to a reduced incidence of PVL and pseudoaneurysms in patients with cardiac Behçet disease and potentially could offer benefits for patients with fragile tissues, such as those with infective endocarditis. Specifically, if the aortic annulus is extremely fragile or destroyed, the issue can be addressed by positioning the graft into the right ventricular outflow tract muscle rather than the aortic annulus and securing it with a continuous suture.^5^ This modification appears to be particularly beneficial in managing patients with bleeding risks, such as those with thrombocytopenia or chronic kidney disease, in whom minimizing perioperative bleeding is critical. Additionally, PVL, a common complication in conventional RDV systems, was not observed in our cases.^7^

Another potential advantage of the modified bio-Bentall operation is its ability to reduce surgical time, which may be particularly beneficial for high-risk patients with compromised cardiac function. The median EF in this study was 46% (range, 28%-60%), and these values were overestimated in the presence of severe AI. Minimizing surgical stress is crucial for such patients, and the streamlined techniques used in this procedure could offer significant advantages. In conventional techniques, the graft itself may serve as a hindering obstacle during the root suturing process, complicating the procedure. However, the modified bio-Bentall operation appears to address this issue by inverting the graft, eliminating structural interference, and potentially facilitating more efficient suturing (Figure 1, *A*). In addition, suturing the coronary buttons before valve insertion may prevent hindrance from the valve strut during suturing of the coronary buttons, allowing for a more streamlined surgical workflow (Figure 1, *B*). The median aortic cross-clamping time was 73 minutes (range, 46-154 minutes). For isolated Bentall operations performed via mini-sternotomy (n = 3), the median aortic cross-clamping time and cardiopulmonary bypass times were 62 minutes and 88 minutes, respectively, notably shorter than those reported in previous studies.^8^^,^^9^ The conventional technique requires assembly of the prosthetic valve and graft during the cardiac ischemic period. Although the recently commercialized KONECT RESILIA aortic valved conduit (Edwards Lifesciences) is preconstructed and could serve as an alternative, this device remains clinically unavailable in South Korea owing to financial constraints, making the modified bio-Bentall technique an attractive option.

RDV use has been associated with an increased risk of rhythm disturbances, particularly when deep sutures are placed in the aortic subannular structure, combined with the radial force exerted by the expandable RDV frame, which may lead to a higher rate of PPM implantation.^10^ In contrast, our study used a technique in which the graft rather than the subannular structure is sutured, which is expected to minimize the risk of rhythm disturbances compared to conventional RDV techniques. However, in our study, 3 patients (27.3%) required PPM insertion postoperatively. One patient, with a history of 2 prior percutaneous coronary interventions, presented with ischemic cardiomyopathy and cardiac Behçet disease, complicated by worsening heart failure, severe AI, and an EF of 35%. An urgent modified bio-Bentall procedure with hemiarch replacement and CABG was performed. Postoperatively, a PPM was placed owing to a complete AVB. Considering this patient's underlying cardiac Behçet disease, the development of AVB can be attributed to the disease's impact on the cardiac conduction system. Another patient underwent surgery for acute type A aortic dissection. Postoperatively, the patient developed complete AVB, necessitating the placement of a PPM. In the absence of an underlying condition that would directly contribute to AVB, a potential association between RDV use and the onset of AVB is suspected. The other patient had underlying tachy-brady syndrome, which eventually necessitated PPM implantation. Given this underlying condition, the association between RDV use and the need for PPM insertion appears to be minimal.

These findings suggest that the PPM insertion rate in this study might be overestimated. In addition, based on the surgical mechanism, the risk of complete AVB following the modified Bentall procedure with RDV implantation is likely to be low. Further experience and larger studies are needed to determine the true incidence of AVB associated with this technique.

This study has some limitations. As a single-center study based on a single surgeon's experience, the findings might not be generalizable. Additionally, the follow-up duration was limited, and the sample size was relatively small.

## Conclusions

Our initial experiences with the modified bio-Bentall operation using an RDV have demonstrated favorable early outcomes in relatively high-risk candidates. These results should be further validated with larger datasets and long-term follow-up, and verified by broader clinical experience.

## Conflict of Interest Statement

The authors reported no conflicts of interest.

The *Journal* policy requires editors and reviewers to disclose conflicts of interest and to decline handling or reviewing manuscripts for which they may have a conflict of interest. The editors and reviewers of this article have no conflicts of interest.

## Supplementary Data

Video 1 Step-by-step surgical procedure for a modified bio-Bentall operation with hemiarch replacement in a 75-year-old male patient with severe aortic insufficiency and annuloaortic ectasia. Video available at: https://www.jtcvs.org/article/S2666-2507(25)00147-6/fulltext.
