# Supplementary figures and images for: Modified bio-Bentall operation with a rapid deployment valve
Source: JTCVS Tech. 2025 Apr 30;32:47–52. doi: 10.1016/j.xjtc.2025.04.003 (PMC12347281; doi:10.1016/j.xjtc.2025.04.003)

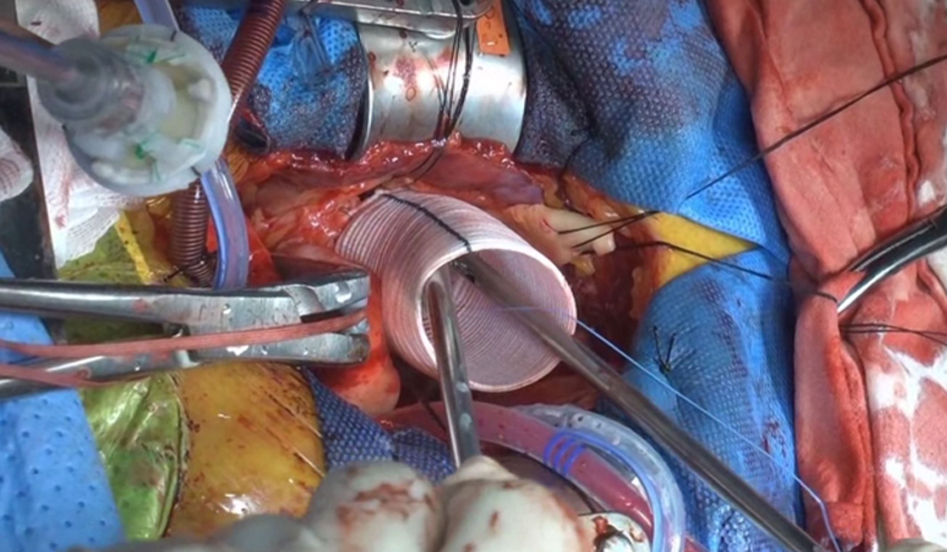

Supplement: Video 1 — Step-by-step surgical procedure for a modified bio-Bentall operation with hemiarch replacement in a 75-year-old male patient with severe aortic insufficiency and annuloaortic ectasia. Video available at: https://www.jtcvs.org/article/S2666-2507(25)00147-6/fulltext. [file fx2.jpg]
